# Eye movement abnormalities in autism spectrum disorder and prodromal psychosis: a review of overlaps and biomarkers

**DOI:** 10.3389/frcha.2026.1758514

**Published:** 2026-03-12

**Authors:** Ethan Terman, Lior Sanilevich, Christian Zaballos

**Affiliations:** Graduate School of Arts and Sciences, New York University, New York, NY, United States

**Keywords:** antisaccade task, autism spectrum disorder, clinical high-risk psychosis, diagnostic specificity, eye-tracking biomarkers, oculomotor dysfunction

## Abstract

**Objectives:**

Differentiating prodromal psychosis from autism spectrum disorder (ASD) during adolescence remains a major clinical challenge due to overlapping behavioral and cognitive features and the absence of reliable biological markers. This review synthesizes evidence on eye tracking and oculomotor abnormalities to evaluate their potential utility as objective biomarkers for distinguishing ASD, clinical high-risk (CHR) states, and prodromal psychosis in adolescent populations.

**Methods:**

A narrative review with structured systematic elements was conducted following PRISMA 2020 reporting principles. Searches were performed in PubMed and PsycINFO, supplemented by citation tracking. Eligible studies included peer-reviewed empirical investigations using eye tracking paradigms in adolescents with ASD, CHR/ prodromal psychosis, or schizophrenia-spectrum conditions. Findings were synthesized narratively due to methodological heterogeneity.

**Results:**

Forty-seven studies were included in the final narrative synthesis. Across paradigms, individuals with ASD demonstrated reduced saccade accuracy, increased endpoint variability, and atypical gaze allocation, often reflecting cerebellar and brainstem dysfunction. CHR and prodromal psychosis samples showed elevated antisaccade error rates, intrusive saccades during smooth pursuit, reduced pursuit gain, and increased fixed entropy, consistent with fronto-striatal and cortico-cerebellar dysregulation. While some oculomotor abnormalities overlapped across groups, schizophrenia-spectrum and CHR samples generally exhibited greater impairment severity and task-related disorganization than ASD alone. However, specificity was limited, and similar abnormalities were observed across other neuropsychiatric conditions.

**Conclusion:**

Eye tracking metrics capture neurodevelopmentally relevant abnormalities in visual attention and oculomotor control that may complement existing clinical assessments of psychosis risk in adolescents with ASD. Current evidence supports their value as research and monitoring tools rather than standalone diagnostic markers. Longitudinal, developmentally stratified studies are required to establish predictive validity, disorder specificity, and clinical translation.

## Introduction

Accuracy in medical diagnostics is crucial for the effective identification, intervention, and treatment of diseases. However, certain conditions, such as prodromal psychosis, present significant challenges due to symptom overlap with other disorders, notably ASD. Understanding these overlaps is essential, as it provides the best opportunity for implementing effective treatment strategies and mitigating the progression to chronic psychosis (1). The two main goals of early intervention in psychotic disorders are to reduce the period of time between the onset of psychosis and the commencement of effective treatment, and to provide consistent and comprehensive care during the critical early years of illness (2).

Accurate identification of the prodromal phase can modify treatment outcomes by potentially enabling timely intervention (1). However, many patients are identifiable only after experiencing symptoms for extended periods, often months or years, which complicates efforts to reduce the duration of untreated psychosis (1). Compounding this issue is the complexity of differentiating prodromal psychosis from other neurodevelopmental disorders, chiefly as ASD. Both conditions share overlapping symptoms, including difficulties with emotional regulation, cognitive impairments, impaired social interactions, and motor skill deficits (3). This overlap creates significant challenges in diagnosis, as clinicians must navigate the nuanced distinctions between prodromal psychosis and ASD, both of which can present with similar symptomatology.

**Figure 1:**
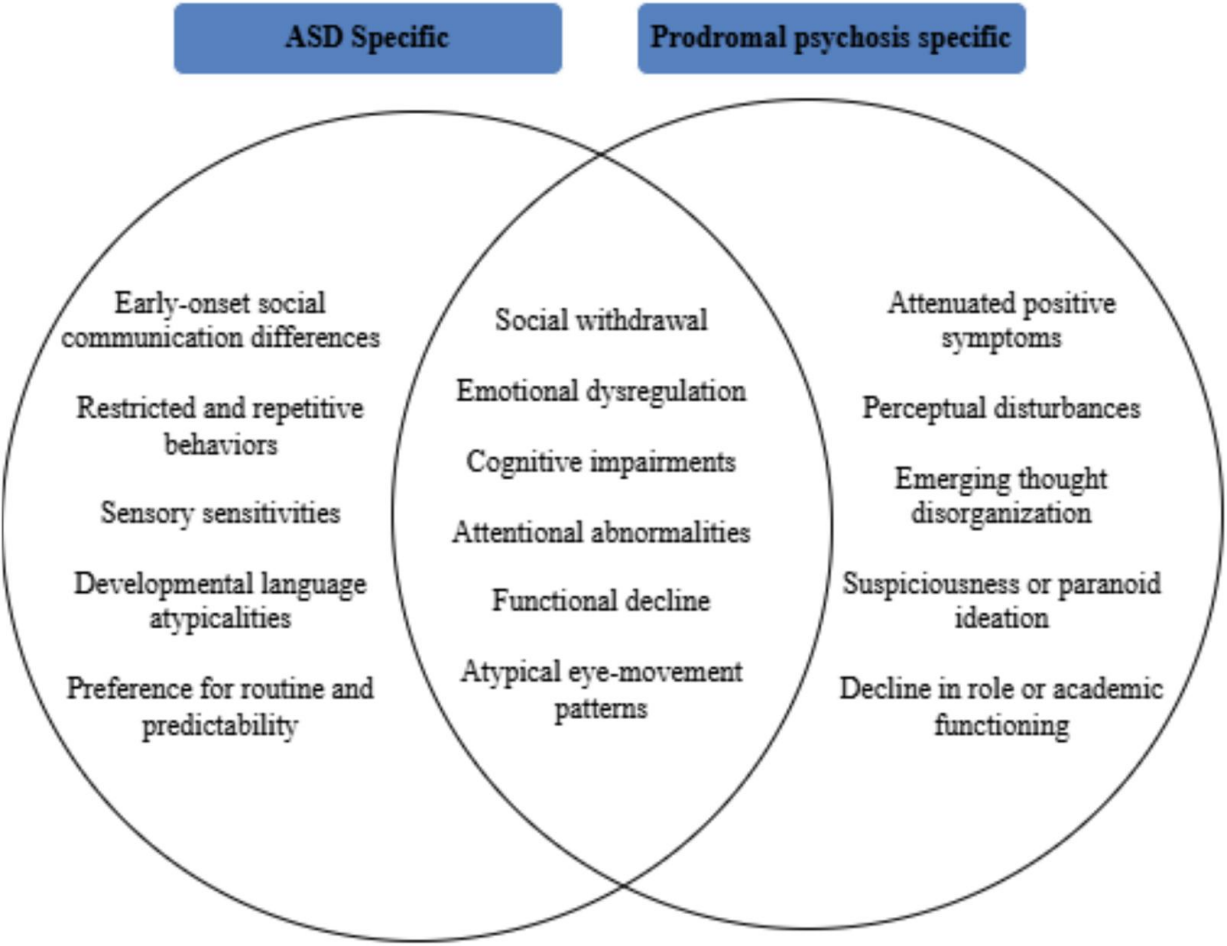
Conceptual venn diagram. Conceptual Venn diagram illustrating overlapping and distinct clinical features of ASD and prodromal psychosis in adolescence. Symptom domains are synthesized from prior diagnostic and clinical literature and are intended to highlight sources of diagnostic ambiguity rather than prevalence or severity differences (8, 9).

Prodromal individuals are often adolescents and young adults experiencing mild or moderate disturbances in perception, cognition, language, motor function, will, initiative, level of energy and stress tolerance (4). 10%–20% of individuals experience sudden psychotic symptoms without a notable prodromal phase (5). A need for advanced diagnostic tools to understand overlaps between prodromal psychosis and ASD is important for treatment planning (6). Eye tracking technology has been investigated as a tool in this context, offering the potential to enhance diagnostic accuracy by providing objective, real-time measures of eye movements. Specifically, smooth-pursuit eye tracking has been proposed as a genetic marker for prodromal psychosis due to its association with abnormalities in eye movement patterns, such as catch-up saccades and difficulties in suppressing anticipatory saccades (7). This technology has the potential to differentiate between ASD and prodromal psychosis by revealing specific eye movement abnormalities that may be indicative of emerging psychotic symptoms. Further research is needed to validate the efficacy of eye tracking and to refine its application as a diagnostic and research tool. Addressing this diagnostic overlap is crucial for improving assessment and treatment planning in adolescents. Eye tracking technology has the capacity to be a useful tool in this regard, offering the prospect of improved diagnostic precision. As research in this area progresses, it may lead to significant advancements in early detection and management, potentially allowing researchers to better study individuals at risk for psychotic disorders. This review explores potential associations between eye movement abnormalities in each disorder and these overlaps. As illustrated conceptually in Figure 1, overlapping domains such as social withdrawal, atypical gaze patterns, and cognitive rigidity contribute to diagnostic ambiguity between ASD and psychosis-risk states.

### Understanding autism spectrum disorder

Historically, ASD was often conflated with schizophrenia-spectrum diagnoses, reflecting longstanding diagnostic ambiguity between neurodevelopmental and psychotic presentations (10, 11). Eye-tracking systems quantify visual behavior by measuring fixation duration, saccadic accuracy, pursuit gain, scan path variability, and latency metrics. These measures provide objective indices of oculomotor control and attentional allocation (12–17). Eye tracking technology has been investigated as an approach for examining candidate biomarkers in relation to prodromal psychosis in individuals with ASD. Eye tracking has been investigated as an approach for examining candidate biomarkers relevant to prodromal psychosis in ASD. Because both conditions involve atypical gaze allocation and attentional regulation, oculomotor metrics may help characterize group-level differences and developmental trajectories (18).

Advanced eye-tracking techniques carry the potential to facilitate research on prodromal psychosis and ASD (18). Metrics such as fixation entropy and pupil dilation may provide additional information relevant to group-level differentiation, supporting more nuanced investigation of overlapping features (1). Fixation entropy measures the variability and randomness in eye movement patterns, which can signal cognitive disorganization, a hallmark of the prodromal phase of psychosis. Higher entropy may indicate difficulties in organizing and processing information, often seen in early psychosis (19). Similarly, pupil dilation may reflect insights into cognitive and emotional states; increased dilation often reflects heightened mental workload or stress, which are associated with the onset of psychotic symptoms. Monitoring these metrics can help differentiate between typical variations in eye movements due to ASD and those suggesting the onset of psychosis (8, 20). By capturing real-time data on visual and cognitive processing, researchers can track changes in gaze patterns over time, providing a dynamic assessment of symptom progression (20). This continuous monitoring can be particularly valuable for researchers investigating comorbidities with ASD, where conventional diagnostic criteria might be less effective due to overlapping symptoms and atypical presentations.

### Diagnostic approaches for autism spectrum disorder and prodromal psychosis

#### Diagnosis of autism spectrum disorder

Diagnosis of autism spectrum disorder (ASD) is ultimately clinical and based on established diagnostic criteria, including DSM-5-TR and ICD-11 frameworks (21–23). Structured instruments such as the Autism Diagnostic Observation Schedule, Second Edition (ADOS-2), and the Autism Diagnostic Interview–Revised (ADI-R) serve as standardized assessment tools that support, but do not replace, clinical judgment (24, 25). These instruments assess core domains of social communication and restricted or repetitive behaviors that define ASD (21). Although widely used across diverse populations, diagnosis remains behaviorally based, as no reliable biological markers currently exist to objectively confirm ASD (21–23, 26).

#### Diagnosis of prodromal psychosis

Clinical high-risk (CHR) or prodromal psychosis states are identified using structured instruments such as the Structured Interview for Prodromal Symptoms (SIPS) and the Comprehensive Assessment of At-Risk Mental States (27, 28). Early symptoms, including attenuated psychotic experiences, social withdrawal, and cognitive changes, are often subtle and nonspecific, complicating differentiation from typical developmental variation or other psychiatric conditions (29). As with ASD, diagnosis relies primarily on behavioral evaluation and clinical interpretation in the absence of validated biological markers (30–32).

### Key differences and diagnostic challenges

ASD is conceptualized as a neurodevelopmental condition with early onset, whereas CHR represents a risk state characterized by emerging attenuated psychotic symptoms and heterogeneous trajectories. Despite differences in developmental timing and symptom course, both frameworks depend heavily on behavioral assessment and are challenged by overlapping features such as social withdrawal, attentional variability, and cognitive differences. Additional factors, including variability in symptom presentation (33) and sociocultural influences on symptom reporting and interpretation (34), may further complicate timely identification. Together, these shared limitations highlight the need for complementary objective approaches to improve diagnostic clarity in adolescents.

### Current diagnostic landscape and the diagnostic utility of novel biomarkers

#### Imaging studies

The following studies are included to contextualize mechanistic interpretations of oculomotor findings and were not considered diagnostic evidence within the primary synthesis. Longitudinal imaging research has identified progressive gray matter reductions in medial temporal, cingulate, frontal, and cerebellar regions among individuals who later develop psychosis (35). Similar patterns of cortical and subcortical abnormalities have been reported across psychosis-spectrum disorders, with structural changes more closely associated with global cognitive dysfunction than with specific diagnostic categories (36). These findings support neurodevelopmental models of psychosis and provide a biological context for interpreting transdiagnostic oculomotor abnormalities.

#### Biomarkers

In the context of distinguishing prodromal psychosis from ASD in adolescence, oculomotor metrics have been examined as candidate neurocognitive markers. Traditional diagnostic approaches rely on clinical interviews and behavioral observation, which may be limited by symptom overlap across conditions (3). Eye-tracking paradigms quantify saccadic accuracy, antisaccade inhibition, smooth pursuit eye movements (SPEM), fixation entropy, and pupil dilation, providing objective indices of attentional control and motor regulation (12).

In ASD, studies report reduced saccade accuracy, increased endpoint variability, and atypical gaze allocation, with some evidence suggesting developmental differences in oculomotor maturation (37). In contrast, prodromal psychosis and clinical high-risk (CHR) states are more consistently associated with elevated antisaccade error rates, intrusive saccades, altered scan paths, and reduced fixation durations (38–40). Comparative findings indicate that while schizophrenia-spectrum groups often show more severe saccadic disorganization, ASD patterns tend to be intermediate or context-dependent, limiting clear qualitative separation between conditions (38, 39). SPEM abnormalities, including reduced pursuit gain and increased corrective saccades, are robustly replicated in schizophrenia-spectrum and high-risk samples and have been linked to genetic vulnerability and transition risk (41–43). However, similar though typically less pronounced pursuit differences have also been observed in ASD, reinforcing the transdiagnostic nature of these findings.

Advanced metrics such as fixation entropy and pupil dilation have been investigated as additional indices of cognitive disorganization and arousal regulation (44, 45). While these measures may enhance sensitivity at the group level, their specificity remains uncertain. Across studies, oculomotor abnormalities appear to differentiate ASD and psychosis risk primarily by severity and task-dependent disorganization rather than by distinct disorder-specific signatures. Limited sample sizes, developmental heterogeneity, and cross-sectional designs further constrain their diagnostic utility when used in isolation.

## Methods

### Design and review framework

This manuscript is a narrative review with structured systematic elements conducted to synthesize and critically evaluate published research on eye tracking and oculomotor abnormalities relevant to ASD, prodromal psychosis, and related high-risk states in adolescents. The review followed PRISMA 2020 guidelines where applicable to narrative reviews, with transparent reporting of search strategies, eligibility criteria, and study selection processes. Given the multi-year development of this project and its narrative synthesis design, PRISMA guidance was applied retrospectively to document the literature identification and selection process rather than as a prospectively registered systematic review.

### Information sources and search strategy

A comprehensive search was conducted in PubMed and PsycINFO to capture three overlapping streams of literature: (1) ASD and eye-tracking, (2) ASD, psychosis-risk, and eye-tracking, and (3) psychosis-risk or schizophrenia and eye-tracking. Search terms included controlled vocabulary (e.g., MeSH terms in PubMed) and free-text keywords. Core terms were combined using Boolean operators and included variations of the following concepts: *autism spectrum disorder*, *prodromal psychosis*, *clinical high-risk*, *ultra-high-risk*, *schizophrenia*, *eye tracking*, *oculomotor*, *saccadic eye movements*, and *smooth pursuit*. Searches were limited to peer-reviewed articles published in English between 2000 and 2026, reflecting the period of substantial methodological development in eye-tracking research. Although the primary focus of this review was on empirical studies published from 2000 onward, seminal historical works were included selectively to provide necessary theoretical and diagnostic context. In addition to database searches, citation tracking of key review articles and seminal empirical papers was conducted manually to identify additional relevant studies. Google Scholar was used solely as a supplementary citation-tracking tool and not treated as an independent database.

### Eligibility criteria

Studies were eligible for inclusion if they met the following criteria:

Population: Included adolescents or youth samples with a mean age between approximately 10 and 19 years, or mixed-age samples in which adolescent data were reported separately. Studies focusing exclusively on adults or early childhood were excluded unless findings were directly relevant to developmental trajectories.
Clinical Groups: Included participants diagnosed with ASD, individuals at clinical high-risk for psychosis (e.g., prodromal, ultra-high-risk, or at-risk mental state), or schizophrenia-spectrum conditions.
Methodology: Employed eye-tracking or oculomotor paradigms (e.g., saccades, smooth pursuit, fixation, gaze patterns) as a primary outcome measure.
Study Type: Empirical studies reporting original data. Reviews, theoretical papers, and commentaries without new data were excluded.
Publication Status: Peer-reviewed, published articles only.

Neuroimaging methods (e.g., fMRI, structural MRI) were not required for inclusion; however, studies integrating eye-tracking with neuroimaging were retained when eye-tracking outcomes were reported.

### Study selection process

All records retrieved from database searches were exported and duplicates removed prior to screening. Titles and abstracts were screened for relevance based on the eligibility criteria. Full-text articles were then assessed for inclusion. The screening and selection process was iterative and involved independent review by the primary author, with eligibility decisions guided by predefined criteria. Ambiguous cases were resolved through closer examination of study methods and sample characteristics. Reasons for exclusion at the full-text stage were documented. A total of 1,905 records were identified through database searching, with an additional 200 records identified through citation tracking. After duplicate removal, 1,600 records underwent title and abstract screening. Of these, 180 articles were assessed at the full-text level. Ultimately, 47 studies met the inclusion criteria, with 47 studies retained for the final narrative synthesis due to direct relevance to adolescent populations and eye-tracking outcomes.

### Data extraction and synthesis

For each included study, the following information was extracted: eye-tracking paradigm, key oculomotor measures, main findings, and reported limitations. Given heterogeneity in paradigms, outcomes, and populations, a quantitative meta-analysis was not feasible. Instead, findings were synthesized narratively and organized thematically. To clearly separate foundational context from study-derived evidence, all sections following the Methods that are labeled “Results” summarize findings extracted from the 47 included empirical studies.

### Critical appraisal

**Figure 2:**
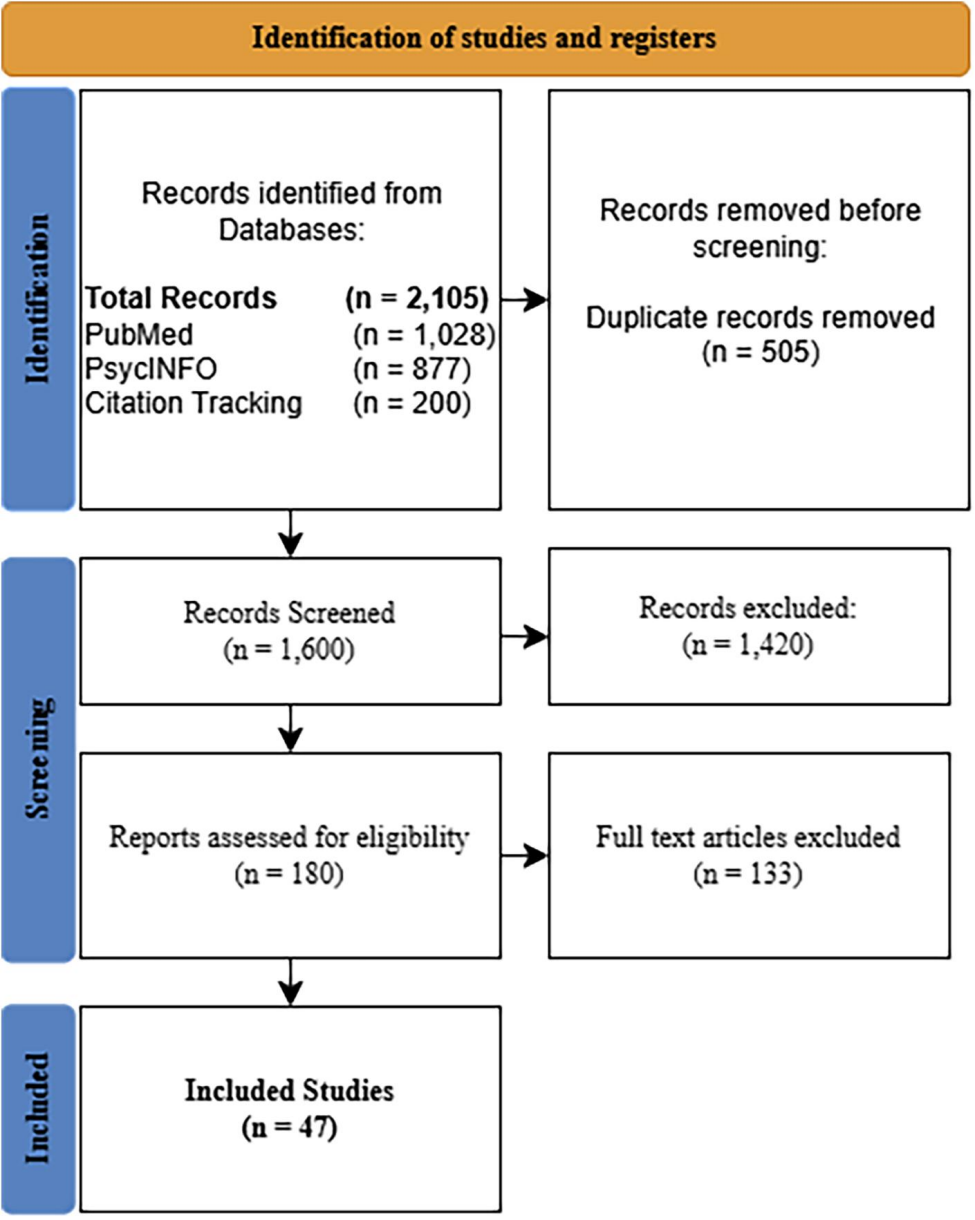
PRISMA flow diagram. PRISMA flow diagram illustrating study identification, screening, eligibility, and inclusion.

Rather than formal risk-of-bias scoring, methodological strengths and limitations were evaluated qualitatively across studies, with attention to sample size, diagnostic rigor, developmental specificity, task validity, and reproducibility of eye-tracking metrics. Conflicting findings and methodological constraints were explicitly addressed in the synthesis to provide a balanced and critical assessment of the literature. The study identification and selection process is summarized in Figure 2.

## Results

### Study characteristics and qualitative synthesis of included studies

The following section synthesizes findings exclusively from the 47 included studies, organized by diagnostic groups to clarify patterns of overlap and divergence. A total of 1,905 records were identified through database searching and 200 additional records through citation tracking. After duplicate removal and screening, 47 studies met inclusion criteria and were retained for narrative synthesis based on direct relevance to adolescent populations and eye-tracking outcomes. The included literature was heterogeneous in task paradigms, eye-tracking metrics, clinical samples (ASD, clinical high-risk/prodromal psychosis, schizophrenia-spectrum, and control groups), and analytic approaches. Accordingly, findings were synthesized qualitatively and organized by paradigm domain and clinical group.

Across ASD adolescent studies, the most consistently reported oculomotor differences involved reduced saccade accuracy and increased endpoint variability, suggesting imprecision in oculomotor calibration rather than a uniform alteration in mean latency across paradigms (37). In visually guided saccade tasks, ASD samples commonly showed greater absolute endpoint error and/or increased trial-to-trial variability relative to controls (37). Variability in effect magnitude across studies likely reflected differences in age composition, target parameters, and analytic definitions. Overall, the ASD evidence supports oculomotor imprecision/variability as a repeatable feature in adolescence, while underscoring that specific metric sensitivity varies by paradigm.

Across free-viewing and scene exploration paradigms, ASD studies also reported atypical gaze allocation patterns relative to controls, although the direction and magnitude of effects varied across stimulus types (social vs. non-social), region-of-interest definitions, and task instructions (38). Some paradigms emphasized social attention (e.g., distribution of gaze to socially salient regions), whereas others evaluated non-social exploration; heterogeneity in stimuli and analytic pipelines limited direct cross-study comparability. Accordingly, the ASD evidence supports atypical gaze allocation as a recurring group-level feature while also indicating meaningful context-dependence that constrains a single “signature” gaze phenotype across paradigms.

Among clinical high-risk/prodromal psychosis and schizophrenia-spectrum studies, antisaccade abnormalities were among the most frequently reported and robust findings, typically reflected in elevated antisaccade error rates and impaired inhibitory control/goal maintenance under conditions requiring suppression of a reflexive saccade (38–40). Antisaccade performance was often discussed as a candidate vulnerability marker within the psychosis spectrum; however, evidence for diagnostic specificity was limited by overlap with other neuropsychiatric conditions and by the relative scarcity of direct ASD vs. CHR comparisons using identical paradigms (39, 40). Smooth pursuit paradigms in CHR/prodromal and schizophrenia-spectrum samples frequently demonstrated reduced pursuit gain and increased saccadic intrusions (e.g., catch-up or anticipatory saccades), consistent with tracking instability and altered sensorimotor prediction (41–43, 46). Pursuit deficits were commonly described as detectable prior to full psychosis onset and were discussed as potential markers of risk or vulnerability; however, variability in pursuit task parameters and reporting practices limited inference of a single uniform CHR “pursuit signature.” A subset of studies applied computational features of gaze organization, including entropy-based indices of fixation structure during free-viewing tasks (46). Across psychosis-risk samples, these studies often reported increased fixation entropy or related markers of disorganized exploration, interpreted as less structured visual sampling (45, 47, 48). In ASD samples, advanced computational metrics were less consistently applied, and when reported, interpretation depended strongly on stimulus class and analytic method.

Across the included literature, oculomotor abnormalities were observed in both ASD and psychosis-spectrum samples, supporting transdiagnostic overlap. However, psychosis-spectrum studies more consistently reported greater impairment severity and more prominent abnormalities in antisaccade inhibition and pursuit stability than was typically observed in ASD alone. At the same time, specificity remained limited: similar abnormalities, including antisaccade errors, pursuit instability, and gaze variability, were reported across multiple neuropsychiatric conditions, constraining standalone diagnostic utility. Overall, the evidence supports eye tracking as a complementary biomarker within multimodal developmental frameworks rather than a single discriminative test for differentiating ASD from prodromal psychosis. Across the 47 included studies, no single eye-tracking metric demonstrated consistent disorder-specific specificity sufficient to reliably distinguish ASD from CHR/ prodromal psychosis at the individual level.

## Discussion

Early psychotic symptoms are often subtle, non-specific, and difficult to distinguish from core features of ASD, including social communication difficulties, emotional dysregulation, and cognitive impairments (8, 9, 49). Because the prodromal phase precedes the onset of full psychosis, accurate and timely identification is critical for effective intervention and for mitigating progression to more severe illness (8). However, overlapping symptom profiles between ASD and prodromal psychosis continue to complicate diagnostic decision-making and highlight existing assessment frameworks (29). Eye tracking paradigms have therefore been investigated as a potential objective complement to traditional diagnostic approaches by quantifying oculomotor control and visual attention processes that are not readily captured through clinical interviews or behavioral observation. Traditional diagnostic methods remain foundational but are inherently limited by subjective judgment and reduced sensitivity to subtle neurocognitive differences, particularly in individuals with ASD.

### Integrative synthesis of Eye tracking findings across conditions

**Figure 3:**
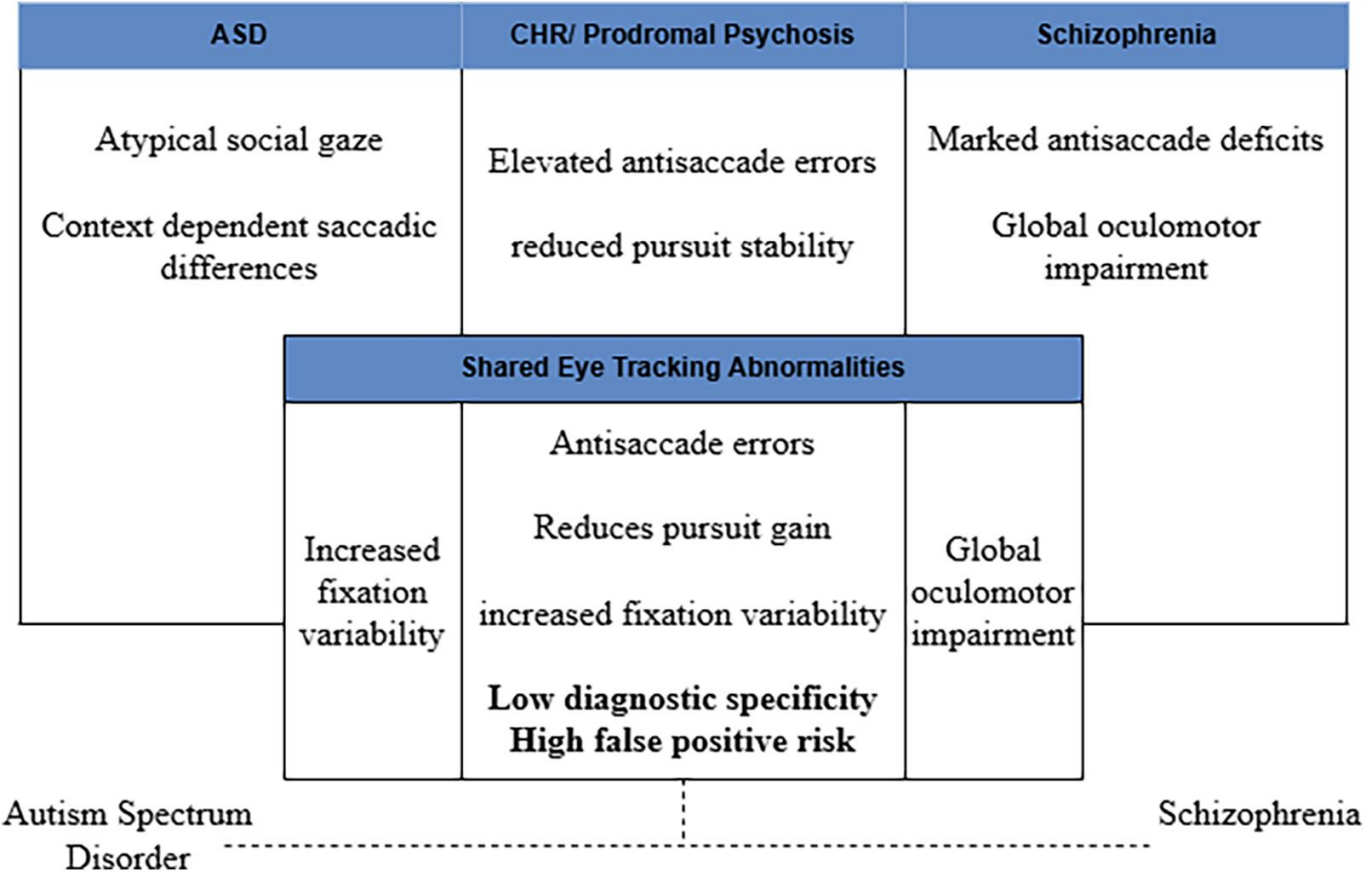
Limited diagnostic specificity of eye tracking measures across autism spectrum disorder and psychosis spectrum conditions. Substantial overlap in eye tracking abnormalities, including antisaccade errors, reduced smooth pursuit gain, and increased fixation variability, is observed across ASD, CHR states, and schizophrenia (38–44, 46). This overlap contributes to low diagnostic specificity and elevated false positive risk when eye tracking metrics are used in isolation. Accordingly, eye tracking measures are best interpreted as complementary biomarkers within multimodal and developmental clinical frameworks rather than as standalone diagnostic tools.

Despite consistent group-level differences in oculomotor performance across schizophrenia-spectrum and high-risk populations, the current evidence base is characterized by small sample sizes, task heterogeneity, and limited longitudinal validation, constraining clinical translation. Notably, few studies directly compare ASD and CHR populations within the same experimental paradigms, limiting inferences about diagnostic specificity and reducing confidence in eye tracking measures as differentiating biomarkers. Findings from schizophrenia-spectrum research are therefore best interpreted as informing developmental and mechanistic models of psychosis vulnerability rather than serving as direct diagnostic proxies for prodromal psychosis in ASD. Abnormalities in antisaccade performance, smooth pursuit eye movements, and fixation dynamics are robustly observed in schizophrenia-spectrum and CHR samples, yet similar patterns, often of lesser severity, are also reported in ASD. Notably, the same oculomotor abnormalities that predict psychosis risk in clinical high-risk populations may inflate false positive risk in ASD when developmental context and baseline neurodevelopmental traits are not explicitly modeled. Figure 3 conceptually illustrates the substantial overlap in eye tracking abnormalities across ASD and psychosis spectrum conditions that underlie this limited diagnostic specificity.

Neuroimaging methods were not required for inclusion in this review and are therefore summarized only as mechanistic context for neural systems plausibly underlying oculomotor control. Unless imaging measures are explicitly analyzed in relation to eye-tracking outcomes within the same study they should not be interpreted as direct evidence for the diagnostic utility of eye tracking. We therefore restrict conclusions about biomarker utility to the eye-tracking phenotypes synthesized in the Results and treat neuroimaging findings as supportive context for potential pathophysiological interpretation.

Despite its promise as an objective and scalable assessment tool, eye tracking technology has several limitations that constrain its clinical utility for differential diagnosis. Eye movement measures are highly sensitive to task design, cognitive load, attentional state, and participant compliance, introducing substantial variability across studies and limiting cross paradigm comparability. Moreover, many commonly reported abnormalities, such as antisaccade errors, reduced smooth pursuit gain, and increased fixation entropy, are transdiagnostic and observed across a range of neurodevelopmental and psychiatric conditions. These factors reduce diagnostic specificity and increase susceptibility to false positive classification, particularly in heterogeneous populations such as adolescents with ASD. Without longitudinal baselines, standardized paradigms, and integration with clinical and developmental data, eye tracking metrics alone are insufficient to reliably distinguish prodromal psychosis from stable neurodevelopmental variation.

Taken together, current evidence supports eye tracking as a complementary research and monitoring tool rather than a standalone diagnostic marker for differentiating prodromal psychosis from ASD. In clinical settings with high ASD prevalence, reliance on eye tracking metrics in isolation may therefore misclassify stable neurodevelopmental traits as prodromal change, underscoring the need for cautious interpretation and multimodal contextualization. However, meaningful clinical translation will require longitudinal, developmentally stratified studies with direct comparative designs, standardized paradigms, and validated outcome measures. Until such evidence is available, interpretive caution remains essential when considering eye tracking biomarkers for clinical decision making in psychosis risk and neurodevelopmental disorders.
